# Bioinformatics-based analysis of the roles of basement membrane-related gene AGRN in systemic lupus erythematosus and pan-cancer development

**DOI:** 10.3389/fimmu.2023.1231611

**Published:** 2023-09-29

**Authors:** Rundong Lv, Lei Duan, Jie Gao, Jigang Si, Chen Feng, Jun Hu, Xiulan Zheng

**Affiliations:** ^1^ Department of Clinical Pharmacy, Zibo Central Hospital, Zibo, Shandong, China; ^2^ Department of Pharmacy, Second Affiliated Hospital of Soochow University, Suzhou, Jiangsu, China; ^3^ Department of Children’s Health, Zibo Central Hospital, Zibo, Shandong, China; ^4^ School of Pharmacy, Faculty of Medicine, Macau University of Science and Technology, Macao, Macao SAR, China; ^5^ Department of Rheumatology and Immunology, The Affiliated Drum Tower Hospital of Nanjing University Medical School, Nanjing, Jiangsu, China

**Keywords:** basement membrane, systemic lupus erythematosus, AGRN, immune infiltration analysis, pan-cancer, type I IFN, parainflammation

## Abstract

**Introduction:**

Systemic lupus erythematosus (SLE) is an autoimmune disease involving many systems and organs, and individuals with SLE exhibit unique cancer risk characteristics. The significance of the basement membrane (BM) in the occurrence and progression of human autoimmune diseases and tumors has been established through research. However, the roles of BM-related genes and their protein expression mechanisms in the pathogenesis of SLE and pan-cancer development has not been elucidated.

**Methods:**

In this study, we applied bioinformatics methods to perform differential expression analysis of BM-related genes in datasets from SLE patients. We utilized LASSO logistic regression, SVM-RFE, and RandomForest to screen for feature genes and construct a diagnosis model for SLE. In order to attain a comprehensive comprehension of the biological functionalities of the feature genes, we conducted GSEA analysis, ROC analysis, and computed levels of immune cell infiltration. Finally, we sourced pan-cancer expression profiles from the TCGA and GTEx databases and performed pan-cancer analysis.

**Results:**

We screened six feature genes (AGRN, PHF13, SPOCK2, TGFBI, COL4A3, and COLQ) to construct an SLE diagnostic model. Immune infiltration analysis showed a significant correlation between AGRN and immune cell functions such as parainflammation and type I IFN response. After further gene expression validation, we finally selected AGRN for pan-cancer analysis. The results showed that AGRN’s expression level varied according to distinct tumor types and was closely correlated with some tumor patients’ prognosis, immune cell infiltration, and other indicators.

**Discussion:**

In conclusion, BM-related genes play a pivotal role in the pathogenesis of SLE, and AGRN shows immense promise as a target in SLE and the progression of multiple tumors.

## Introduction

Systemic lupus erythematosus (SLE) patients have a loss of immune tolerance to autoantigens (e.g., nuclear antigens) in their bodies, which in turn leads to tissue inflammation and multi-organ damage (1). The pathophysiology of SLE is not currently known. Sunlight exposure or viral infection may trigger the disease in genetically susceptible individuals, with the most susceptible group being women of childbearing age (2). Previous studies have demonstrated that immune cells aberrant activation, including B cells (3), T cells (4), macrophages (5), eosinophils (6), and dendritic cells (DCs) (7), plays a significant role in SLE. Notably, SLE patients have a higher risk for overall malignancy (8, 9), which is one of the leading reasons for death in SLE patients (10). Immune system dysregulation could potentially be clinically significant in the development of cancer (11).

The basement membrane (BM) is a cell-adhesive extracellular matrix widely distributed in animal tissues (12) that serves as a supporting junction and a semi-permeable membrane for material permeation (13–16). Research has indicated that genetic defects in BM-related components may result in disease phenotypes in patients, manifesting in various aspects such as the retina (17, 18), kidneys (19), blood vessels (20), skeletal system (21), and muscles (22). BM protein is also the target of self-antibodies in autoimmune diseases (23). Therefore, BM is necessary for maintaining tissue homeostasis in the body and may be closely related to the pathogenesis of SLE and its multi-organ damage. In addition, BM provides clues for cell polarity, differentiation, migration, and survival (15, 24, 25), which significantly contribute to tumor progression, diagnosis, and prognosis (26, 27). Reshaping of BM induces degradation products that play a particularly important role in promoting and inhibiting tumors (28–31). However, the BM-related genes and proteins expression mechanisms in both SLE patients and malignancies have yet to be elucidated. Therefore, it is urgent to explore the BM-related genetic features closely associated with the occurrence and progression of malignancy in SLE patients.

Recently, Ranjay et al. defined an integrated network of BM proteins encoded by human genes (32). Based on this, we applied bioinformatics methods to perform differential expression analysis of BM-related genes in datasets from SLE patients obtained from the GEO database (GSE110169, GSE185047). We utilized LASSO logistic regression, SVM-RFE, and RandomForest to screen for feature genes and construct a diagnosis model for SLE. Subsequently, we performed GSEA analysis, ROC analysis, and calculated the level of immune infiltration by ssGSEA. Finally, we analyzed the relationship between feature genes and immune infiltration. In addition, we comprehensively analyzed the prognostic value of AGRN in cancer patients and assessed the role of AGRN in tumor microenvironment (TME), tumor mutation burden (TMB), and microsatellite instability (MSI). This study aims to clarify the pathological physiology and molecular biology roles of BM-related genes in SLE and multiple tumor development, providing new ideas for diagnostic and personalized therapeutic targets for SLE and cancer.

## Methods

### Download and collation of datasets in SLE

In our study, we downloaded two datasets, GSE110169 and GSE185047, from the GEO database (**Table 1**). We performed array normalization using “limma” in R software. Additionally, we downloaded gene expression data and corresponding clinical data for 33 types of cancer samples from the TCGA database and mRNA expression profiles for 31 different tissues from GTEx for subsequent pan-cancer analysis.

**Table 1:** Information on microarray datasets obtained from GEO.

GEO Dataset	Platform	Samples	Source types	Group
GSE110169	GPL13667	82 SLE patients and 77 Controls	Whole blood	Discovery cohort
GSE185047	GPL570	87 SLE patients and 10 Controls	Whole blood	Validation cohort

### Identification of BM-related genes

Based on previous research reports (32), we extracted the expression of 222 basement membrane genes using the “limma” package and then performed differential expression analysis (*p*.adjust < 0.05 were considered statistically significant). Finally, we used the “pheatmap” package to generate expression heatmaps.

### Functional enrichment analysis

Functional enrichment analysis was performed on BM-related genes with differential expression to confirm potential target functions. The Gene Ontology (GO) was utilized for functional gene annotation, including molecular function (MF), biological process (BP), and cellular component (CC) annotations. KEGG enrichment analysis provides a good reference for functional studies of differentially expressed genes. Additionally, disease ontology (DO) analysis was performed to understand the types of diseases in which BM-related genes are involved.

### Screening for feature genes

Further analysis of differentially expressed BM-related genes will be used to screen for feature genes in SLE. Random Forest and SVM-RFE are two commonly used machine learning methods for selecting key factors. In addition, we perform LASSO regression in order to compute linear models and screen for valuable variables. Finally, the feature genes were determined by taking the intersection set through the Venn diagram.

### Diagnostic model construction and validation

Based on feature genes, we constructed a diagnostic column line graph to forecast the risk of SLE occurrence. The diagnostic model’s prediction accuracy is then evaluated using calibration curve and decision curve analyses. Finally, we used R software to create ROC curves, which were used to screen the highest AUC value of the feature genes for pan-cancer analysis.

### Quantitative real-time polymerase chain reaction

We collected and extracted total RNA from whole blood of 16 SLE patients and 24 healthy individuals. Quantitative real-time polymerase chain reaction (qRT-PCR) was performed to detect mRNA levels. The relative expression of mRNA was normalized to the level of GAPDH. Primers are shown in **Supplementary Table 1**. In addition, each SLE patient's disease activity index score (SLEDAI) was assessed and the correlation between AGRN mRNA levels and SLEDAI was analyzed.

### GSEA analysis

GSEA is a method for interpreting whole-genome expression profiles that can be used to identify the enrichment of different gene sets in specific biological processes. After sorting the feature genes according to their expression patterns, the enrichment score of the gene set in the gene ranking is calculated, and it is visualized using R.

### Immune infiltration analysis by ssGSEA

The degree of immune cell infiltration in the samples was calculated using ssGSEA. To analyze the relationship between immune cells, immune function, and feature genes, the Spearman correlation coefficient was obtained using the “corrplot” package.

### Differential expression analysis of AGRN in tumor tissues

The expression levels of AGRN in 33 tumor tissues were assessed and compared with those of normal tissues. Transform the expression data using log2 conversion and t-test. The expression difference between tumor and normal tissues is based on a standard of *p* < 0.05. Use the “ggplot2” package to create boxplots.

### Immunohistochemistry staining of AGRN

We analyzed the protein expression differences of AGRN using the Human Protein Atlas database (HPA, https://www.proteinatlas.org/). The HPA database provides information on the distribution of proteins in human tissues and cells. We downloaded immunohistochemistry images of tumor tissues and their corresponding normal tissues from the HPA, including breast cancer, liver cancer, lung cancer, prostate cancer, and 12 other tumor types.

### Analysis of AGRN and prognosis of cancer

Analyzing clinical data from the TCGA database, the metrics used to evaluate the correlation between AGRN and the prognosis of cancer patients were overall survival (OS), disease-specific survival (DSS), and progression-free interval (PFI). Perform Kaplan-Meier analysis and plot survival curves using the “survival” and “survivor” packages.

### Relationship between AGRN and immunity

We calculated the stromal score and immune score of the tumor microenvironment by using the “estimate”, “ggplot2,” and “ggpubr” packages. The correlation between AGRN and immune cell infiltration level was explored by the CIBERSORT algorithm. In addition, TMB and MSI analyses were also performed based on the “fmsb” package.

### Statistical analysis

Data are expressed as mean ± standard error (SEM). Unpaired Student’s t-test was used for statistical analysis. Statistical analysis was performed. P-values less than 0.05 were considered statistically significant.

## Results

### Differential expression of BM-related genes in SLE

**Figure 1:**
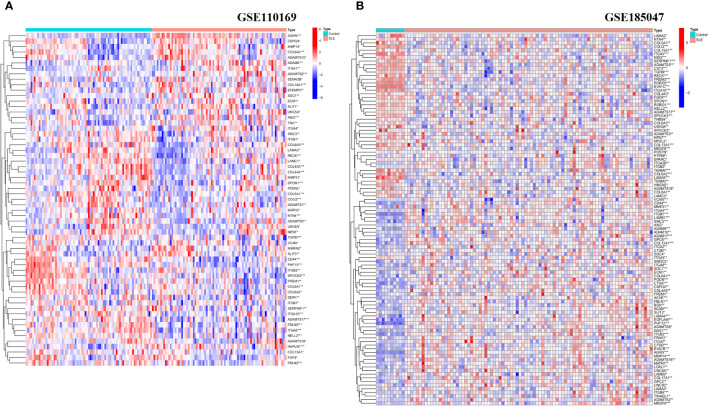
Heatmap analysis of DEBGs in healthy controls and SLE patients. **(A)** Heatmap analysis of Discovery cohort; **(B)** Heatmap analysis of Validation cohort. (*p<0.05, **p<0.01, ***p<0.001).

We analyzed the expression levels of BM-related genes in the healthy controls and SLE group (using *p*-adjustment < 0.05 as the threshold), and the results showed that there were 61 differentially expressed BM-related genes (DEBGs) in the GSE110169 dataset and 102 differentially expressed BM-related genes in the GSE185047 dataset. **Figure 1** shows the heatmap of differentially expressed BM-related genes.

### Functional enrichment analysis

**Figure 2:**
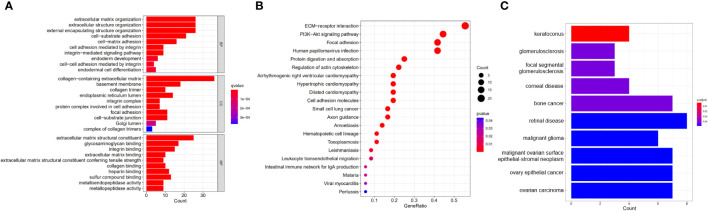
Functional enrichment analysis of DEBGs. **(A)** GO analysis of DEBGs; **(B)** KEGG analysis of DEBGs; **(C)** DO analysis of DEBGs.

Enrichment analysis showed the biological functions connected to the BM-related genes with differential expression. The research findings indicate that the main enriched biological processes include extracellular matrix organization, cell-matrix adhesion, endoderm development, endodermal cell differentiation, integrin binding, sulfur compound binding, and metallopeptidase activity (**Figure 2A**). The KEGG pathways primarily include ECM-receptor interaction, leukocyte transendothelial migration, PI3K-Akt pathway, arrhythmogenic right ventricular cardiomyopathy, cell adhesion molecules, intestinal immunity, and HPV infection (**Figure 2B**). DO analysis is mainly associated with retinal disease, ovarian carcinoma, bone cancer, malignant glioma, corneal disease, glomerulosclerosis, and other diseases (**Figure 2C**).

### Screening of feature genes

**Figure 3:**
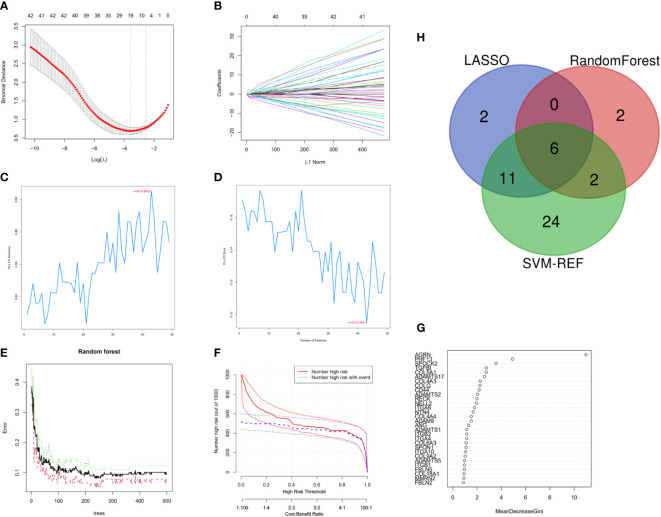
Feature genes selection. **(A)** Selection operator model (lasso); **(B)** Adjustment of feature selection in the minimum absolute shrinkage; **(C, D)** Biomarker signature gene expression validation by SVM–RFE algorithm selection; **(E)** RandomForest error rate versus the number of classification trees; **(F)** Clinical effect plot of random forest model; **(G)** BM-related genes with a MDG value; **(H)** Venn diagram screening for feature genes.

19 predictive genes with statistical significance were selected from univariate data by logistic regression (**Figures 3A, B**); the SVM-RFE results show that the best prediction performance can be obtained by selecting 43 feature variables (**Figures 3C, D**); the combination of random forests and feature selection was used to determine the error rate (**Figures 3E, F**); and the relationship between 30 relatively important genes (**Figure 3G**), with MDG values over 2 for 10 genes. Six overlapping feature genes were identified through a Venn diagram (**Figure 3H**).

### Constructing a diagnostic model for SLE based on feature genes

**Figure 4:**
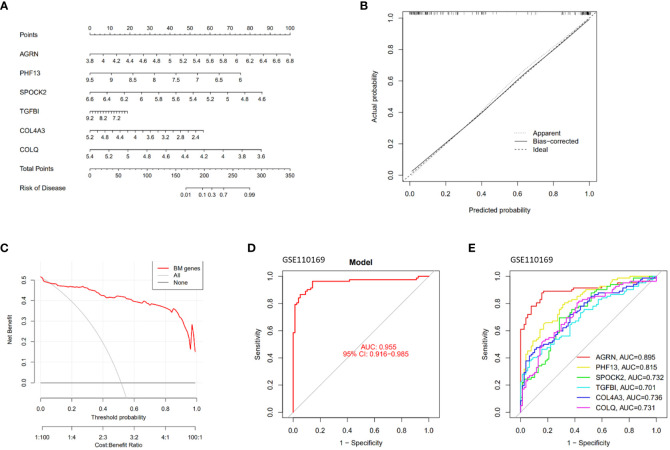
Construction and validation of the SLE diagnostic model. **(A)** Nomogram graphs of SLE; **(B)** Calibration curve of the model; **(C)** Decision Curve Analysis; **(D, E)** ROC curves of the feature genes.

A diagnostic nomogram model for SLE was constructed based on feature genes (AGRN, PHF13, SPOCK2, TGFBI, COL4A3, and COLQ). The R software was employed to visualize the diagnostic nomogram (**Figure 4A**), calibration plot (**Figure 4B**), and decision curve analysis (DCA) (**Figure 4C**) of SLE. The calibration curve and DCA of the diagnostic model demonstrated a good fit with an AUC of 0.955 (**Figure 4D**). The AUC values for the feature genes are presented in **Figure 4E**, with AGRN exhibiting the highest AUC value at 0.895.

### GSEA analysis of the feature genes

**Figure 5:**
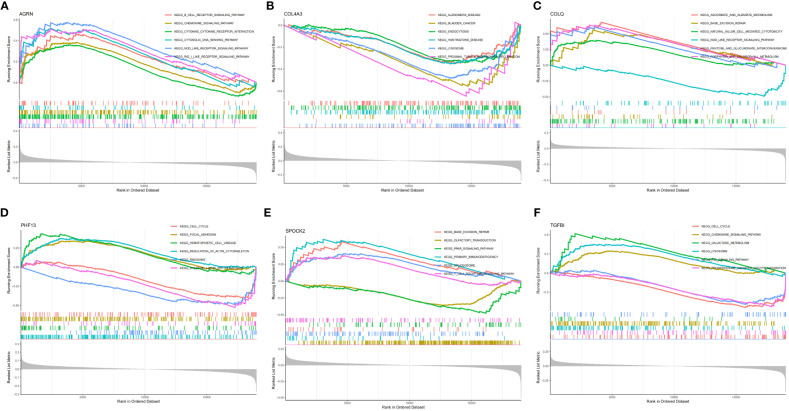
GSEA enrichment analysis of feature genes. **(A)** GSEA analysis of AGRN; **(B)** GSEA analysis of COL4A3; **(C)** GSEA analysis of COLQ; **(D)** GSEA analysis of PHF13; **(E)** GSEA analysis of SPOCK2; **(F)** GSEA analysis of TGFBI.

SLE patients were divided into two groups and analyzed by GSEA based on the median expression values of the feature genes. In the AGRN high-expression subgroup, B cell receptor signaling pathway, chemokine signaling pathway, RIG-I-like receptor (RLR), cytosolic DNA sensing, and NOD-like receptor (NLR) were significantly enriched (**Figure 5A**). Pathways associated with Alzheimer’s disease, bladder cancer, endocytosis, lysosomes, and proximal tubule bicarbonate reclamation were significantly elevated in the COL4A3 low-expression subgroup (**Figure 5B**). In the COLQ high-expression subgroup, pathways such as base excision repair, natural killer cell-mediated cytotoxicity, and porphyrin metabolism were enriched, while in the COLQ low-expression subgroup, the NOD-like receptor signaling pathway was enriched (**Figure 5C**). The PHF13 low-expression subgroup was significantly enriched in ribosomes and systemic lupus erythematosus (**Figure 5D**). In the SPOCK2 high-expression subgroup, pathways such as base excision repair, spliceosome, and T cell receptor signaling were highly enriched, while pathways such as olfactory transduction and PPAR signaling pathway were enriched in the low-expression subgroup (**Figure 5E**). The TGFBI high-expression subgroup was enriched in pathways such as chemokine signaling pathway, lysosome, and galactose metabolism (**Figure 5F**).

### Analysis of immune infiltration

**Figure 6:**
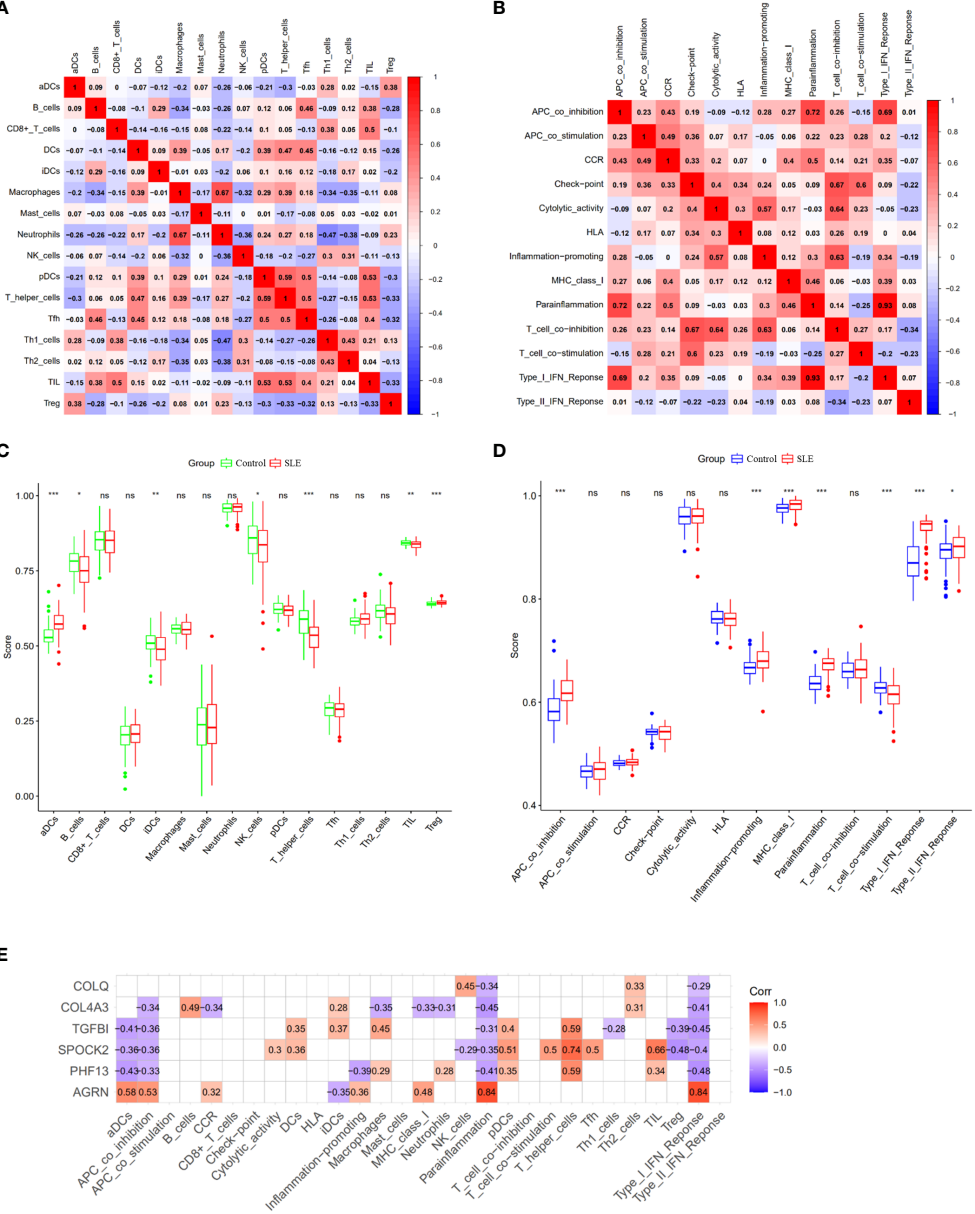
Analysis of ssGSEA immune infiltration. **(A)** Correlation analysis between immune cells; **(B)** Correlation analysis between immune functions; **(C)** Boxplot of differences in immune cell infiltration between SLE and healthy controls; **(D)** Boxplot of immune function differences between SLE and healthy controls; **(E)** Correlation analysis of feature genes with immune function. (ns: no statistical difference, *p<0.05, **p<0.01, ***p<0.001).

Further investigation of the immunological infiltration relationship between SLE and healthy controls was performed by ssGSEA. The correlation analysis of immune cells revealed the existence of multiple pairs of positively and negatively correlated immune cells. Neutrophils and macrophages exhibited a significant positive correlation, while T helper cells and pDCs showed a higher positive correlation. Moreover, Th1 cells were negatively correlated with neutrophils (**Figure 6A**). In terms of immune function correlation analysis, parainflammation and type I IFN Response had a highly positive correlation (**Figure 6B**). The boxplot shows that compared with the healthy controls, aDCs and Treg cells in the SLE group exhibited increased infiltration (*p* < 0.001), while T helper cells, TIL, iDCs, B cells, and NK cells showed decreased infiltration (*p* < 0.05) (**Figure 6C**). In the SLE group compared to the healthy controls, APC co-inhibition, inflammation-promoting, MHC-I, parainflammation, and I-IFN response were all considerably higher (*p* < 0.001) (**Figure 6D**). AGRN was favorably connected with immune cell functions like aDCs, APC co-inhibition, parainflammation, and I-IFN response in a feature gene-immune infiltration correlation study (**Figure 6E**). These feature genes might regulate the immune process as SLE develops. In addition, we conducted the same analysis on the validation queue GSE185047 dataset, and the results showed a consistent correlation between immune cell functions, and a high correlation between AGRN and immune functions such as DCs, parainflammation, and I-IFN response. The results are detailed in **Supplementary Figure 1**.

### Validation of AGRN and diagnostic models

**Figure 7:**
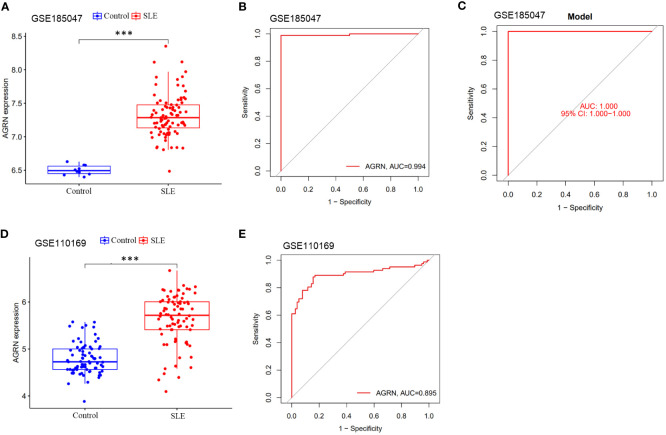
Differential expression boxplot and ROC curve of AGRN. **(A)** Differential expression analysis of AGRN in Discovery cohort; **(B)** ROC curve of AGRN in Discovery cohort; **(C)** Differential expression analysis of AGRN in Validation cohort; **(D)** ROC curve of AGRN in Validation cohort. **(E)** ROC curves of SLE diagnostic column line graph model. (***p<0.001).

We used the GSE185047 dataset to validate the differential expression of AGRN and the accuracy of the diagnostic model. In the validation dataset, AGRN was highly expressed in the SLE group compared to healthy controls (*p* < 0.001) (**Figure 7A**), and the AUC of AGRN was 0.994 (**Figure 7B**). The SLE diagnostic model based on feature genes had an AUC of up to 1.000 (**Figure 7C**), which was consistent with the results obtained from the discovery cohort (**Figures 7D, E**).

### Experimental verification of AGRN expression in whole blood of SLE patients

qRT-PCR analysis showed that peripheral blood AGRN mRNA expression was significantly upregulated in SLE patients (*p*<0.001). The correlation between AGRN levels and systemic lupus erythematosus (SLE) disease activity index score (SLEDAI) was further analyzed. The results showed that AGRN mRNA levels were positively correlated with SLEDAI (R=0.494), but there was no significant difference (*p*=0.052). As shown in **Supplementary Figure 2**.

### Expression of AGRN in pan-cancer

**Figure 8:**
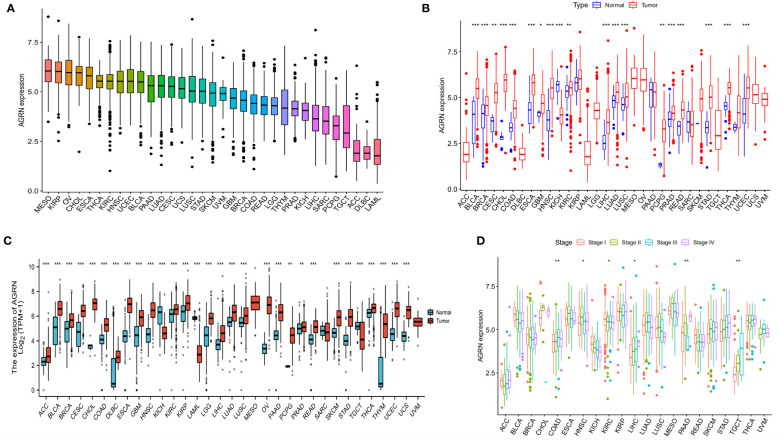
AGRN expression in pan-cancer. **(A)** AGRN expression in cancer cell lines; **(B)** Comparison of AGRN expression between tumor and normal tissue in the TCGA dataset; **(C)** Comparison of AGRN expression between tumor and normal tissue in the TCGA and GTEx datasets; **(D)** Correlation between AGRN expression and pathological staging in the TCGA database. (*p<0.05, **p<0.01, ***p<0.001).

Combining the results of immune infiltration analysis and ROC analysis, we conducted a pan-cancer analysis of AGRN. The analysis showed that various types of cancer expressed AGRN, with the highest level in MESO (**Figure 8A**). AGRN was identified in TCGA data as highly expressed in 18 tumor tissues and lowly expressed in KICH (**Figure 8B**). In addition, analysis of the normal tissue data downloaded from the GTEx database revealed that AGRN expression was elevated in 28 tumor tissues and weakly expressed in KICH and TGCT (**Figure 8C**). In addition, to assess the expression of AGRN at the protein level, we utilized the HPA database to extract immunohistochemistry images. **Supplementary Figure 3** clearly demonstrates that the expression of AGRN protein is significantly higher in tumor tissues, such as breast cancer, cervical cancer, glioma, and lung cancer, compared to normal tissues.

### Prognostic value analysis of AGRN

**Figure 9:**
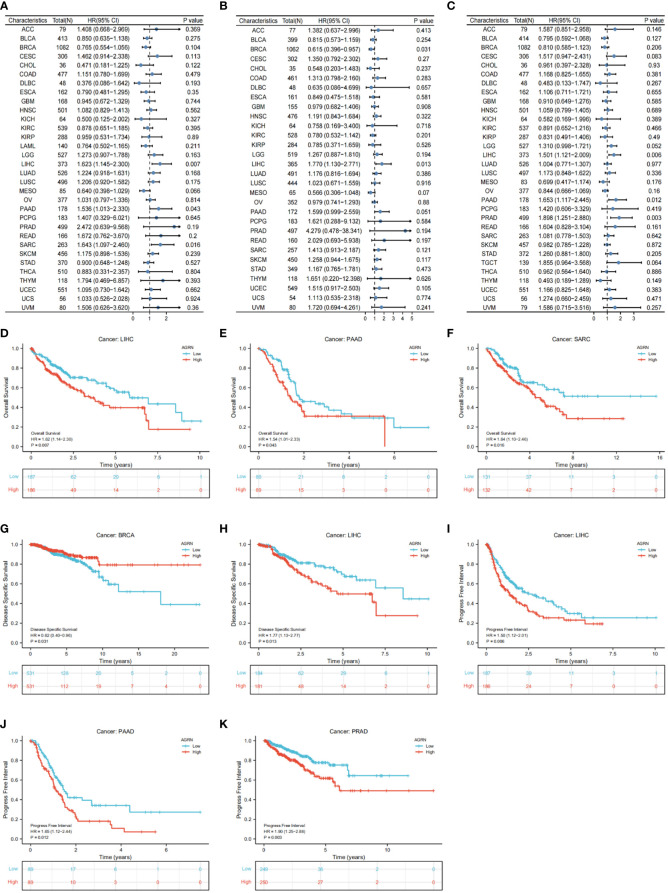
Correlation of AGRN with prognosis in pan–cancer. **(A)** Correlation between AGRN expression and overall survival (OS); **(B)** Correlation between AGRN expression levels and disease-specific survival (DSS); **(C)** Correlation between AGRN expression and PFI; **(D–K)** K-M analysis of the correlation between AGRN expression and OS, DSS and PFI.

We analyzed the relationship between AGRN expression and clinicopathological features in a variety of cancers and found significant associations between AGRN expression in COAD, HNSC, KIRC, LIHC, PAAD, and TGCT at different pathological stages in the TCGA database (**Figure 8D**). Correlation analysis between AGRN expression and survival characteristics showed that high AGRN expression was a high-risk factor for LIHC, PAAD, and SARC (**Figure 9**). In the DSS study, high AGRN expression indicated significantly better prognosis for BRCA (**Figure 9B**), while in LIHC patients, low AGRN expression was inversely related to prognosis. Regarding the association between AGRN and PFS, forest plots and KM survival curves showed that in LIHC, PAAD, and PRAD, patients that expressed more AGRN expression had poorer PFI (**Figure 9C**).

### AGRN and tumor microenvironment

**Figure 10:**
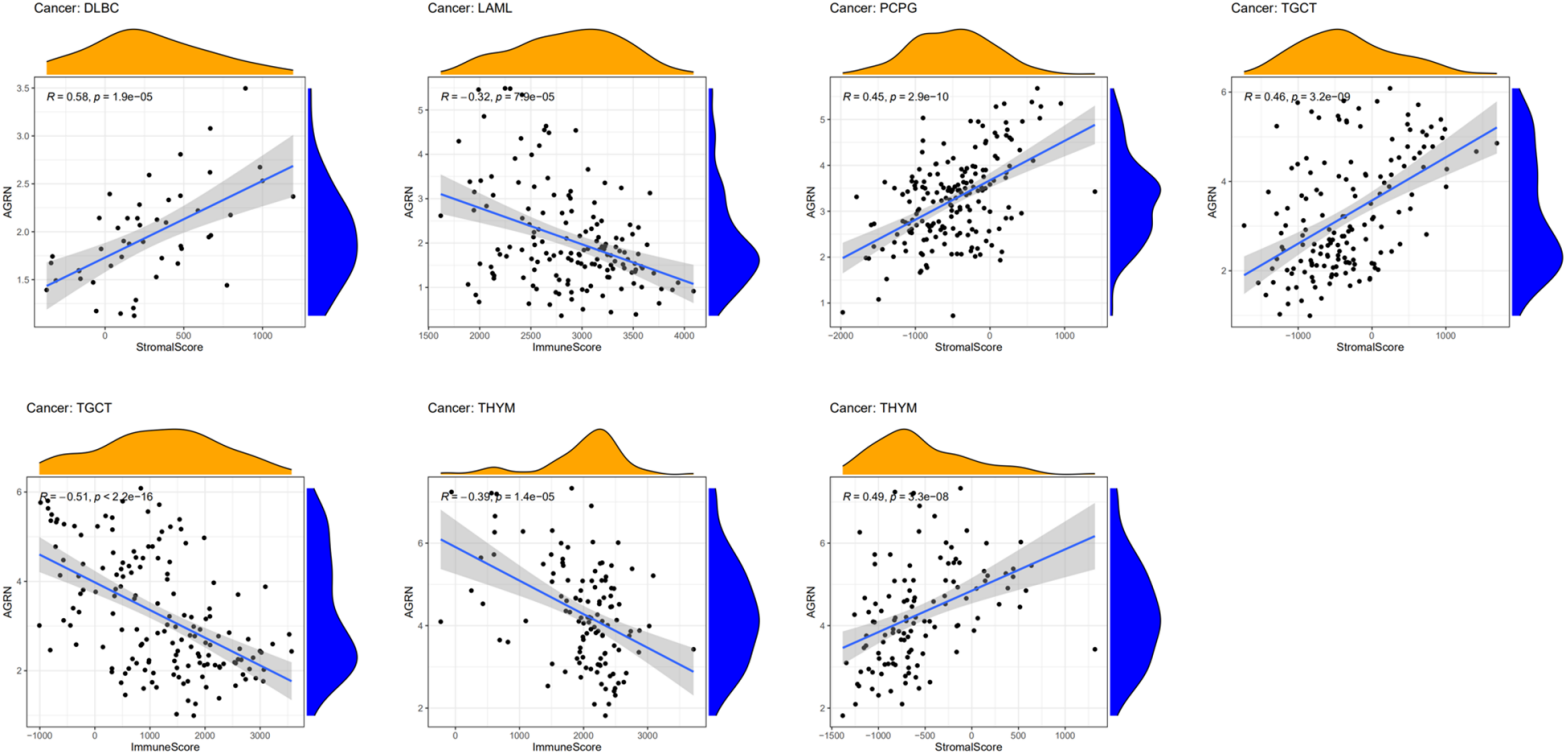
Relationships between AGRN expression and tumor microenvironment.

The tumor immune microenvironment plays an important role in tumor progression (33, 34). We analyzed the relationship between AGRN expression and the tumor microenvironment. The findings showed that AGRN expression was significantly negatively correlated with immune scores of TGCT, LAML, and THYM, while it was significantly positively correlated with stromal scores of DLBC, TGCT, PCPG, and THYM (**Figure 10**).

### AGRN and immune cells infiltration

**Figure 11:**
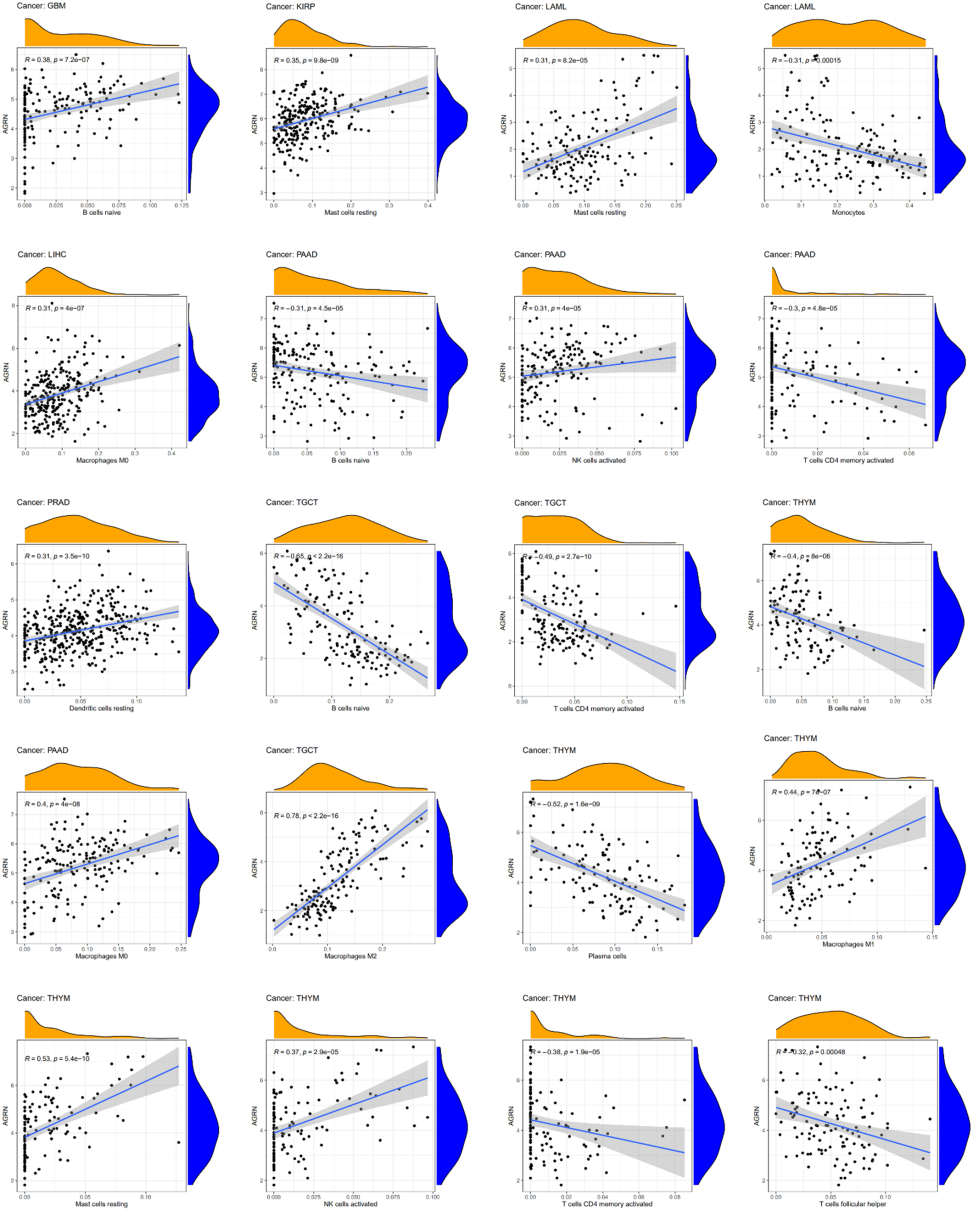
Relationships between AGRN expression and immune cell infiltration.

By analyzing the correlation between AGRN expression and immune cell infiltration in tumor tissues, we found that AGRN expression correlated higher with immune cell infiltration in LAML, TGCT, PAAD, THYM, GBM, KIRP, and LIHC tissues. AGRN was negatively correlated with CD4 T-cell memory, monocytes, plasma cells, and Tfh cells, while it was positively correlated with NK cells activated, mast cells resting, and DCs resting. Interestingly, AGRN expression was positively correlated with multiple subtypes of macrophages (M0, M1, and M2). Additionally, AGRN had a negative correlation with B cell naïve in THYM, PAAD, and TGCT (**Figure 11**).

### The correlation between AGRN and TMB and MSI

**Figure 12:**
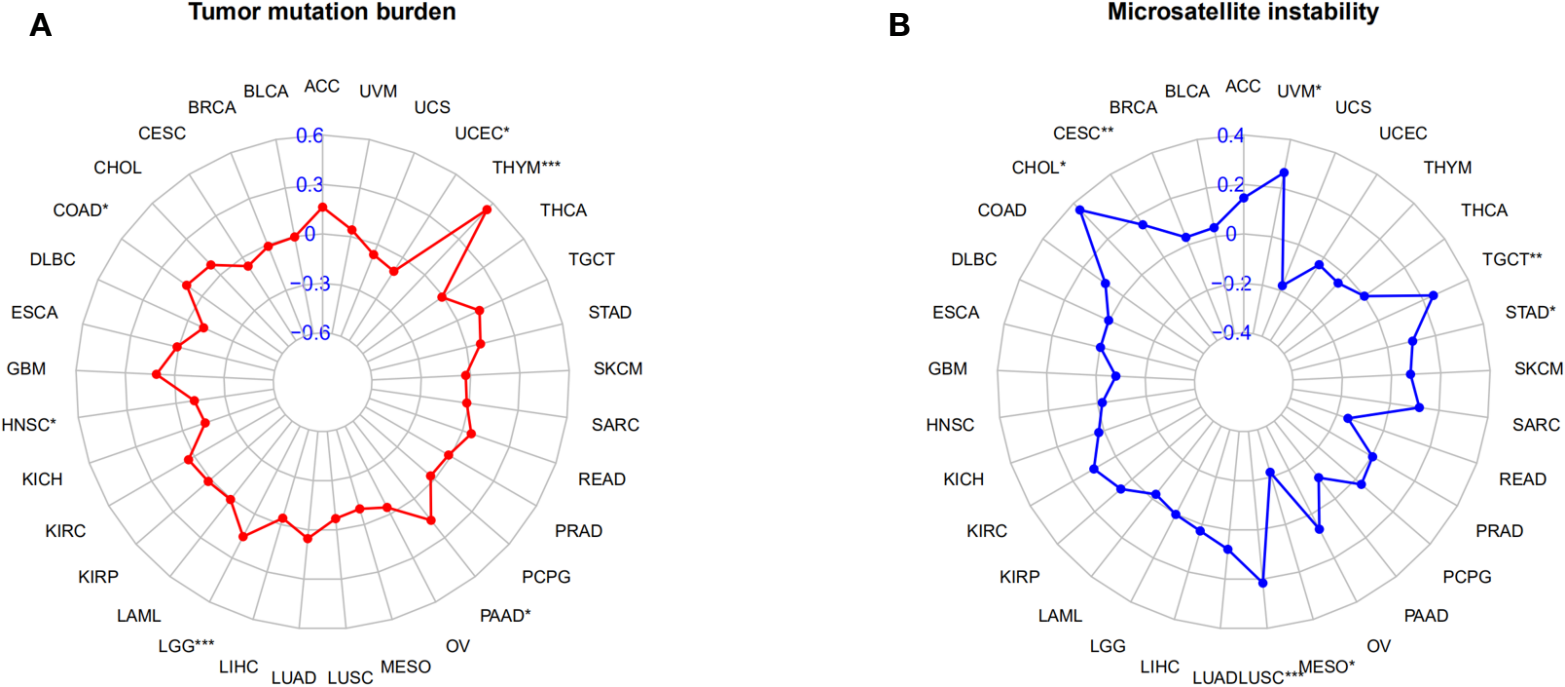
Relationships between AGRN and TMB, MSI. **(A)** Correlation between AGRN and TMB; **(B)** Correlation between AGRN and MSI. (*p<0.05, **p<0.01, ***p<0.001).

In the correlation study of AGRN expression and TMB, AGRN expression in LGG, THYM, UCEC, COAD, HNSC, and PAAD was significantly associated with TMB (**Figure 12A**). In the correlation study of AGRN expression and MSI, AGRN expression in CESC, CHOL, LUSC, MESO, STAD, TGCT, and UVM was significantly associated with MSI (**Figure 12B**).

## Discussion

SLE is an autoimmune disease that affects any organ (35), including the skin, kidneys, nervous system, and heart, and predominates in women between the ages of 20 and 45. Although the mortality rate associated with SLE has decreased with the advancement of research on biologics and stem cell therapies, the pathogenesis of SLE still remains elusive. As a special type of extracellular matrix, BM plays a variety of important functions *in vivo*, such as cell anchoring and signal transduction (36). Research suggests that BM-related components are closely associated with the development of human autoimmune diseases. It has been confirmed in psoriasis that BM destruction is one of the earliest events in its pathogenesis (37). The glomerular BM is a crucial component of the capillary wall in the renal glomerulus, which governs renal filtration (38). Existing studies have shown that the oxidative stress imbalance in lupus nephritis patients can lead to disruption of glomerular BM integrity and affect the renal tubular function of the patients (39). Therefore, exploring the gene and protein expression mechanisms of the BM will provide novel insights into the pathogenesis and therapeutic strategies of SLE.

In this study, we utilized bioinformatics methods to perform expression analysis of BM-related genes in a dataset of SLE patients from the GEO database. We identified 61 differentially expressed BM-related genes. GO enrichment analysis indicated that the differentially expressed BM-related genes were mainly associated with cell-substrate adhesion, metallopeptidase activity, and endodermal cell differentiation. KEGG enrichment analysis revealed a high correlation with ECM-receptor interaction, regulation of the actin cytoskeleton, leukocyte transendothelial migration, and the PI3K-Akt signaling pathway. Subsequently, we employed LASSO regression, SVM-RFE, and RandomForest to screen feature BM-related genes, namely AGRN, PHF13, SPOCK2, TGFBI, COL4A3, and COLQ. The validation dataset confirmed the differential expression of feature genes.

Numerous studies have revealed the relevance of feature genes to autoimmune diseases. Collagen Q (ColQ), a collagen protein commonly found in cholinergic tissues, is involved in the formation of the synaptic basal lamina at neuromuscular junctions. Mutations in this gene are closely related to the occurrence of congenital myasthenic syndrome (40). ColQ is thought to affect acetylcholinesterase through interaction with MuSK (41), and its mutation may lead to myasthenic syndrome. As one of the components that constitute the glomerular BM, defects in COL4A3 can lead to inherited renal diseases (38, 42). In allergic asthma, an increase in the serum level of the COL4A3 degradation marker C4Ma3 is associated with exacerbation of the allergic asthma phenotype, providing a novel biomarker for predicting the response to anti-IgE therapy (43). TGFBI is a protein induced by TGFβ1 and is widely distributed in tissues such as the heart, blood vessels, and eyes (44). Research has shown that in type 1 diabetes, βig-h3/TGF-βi can inhibit T cell activation, effectively preventing the occurrence of autoimmune reactions (45). It is worth noting that TGFBI plays an important role in the diagnosis and pathogenesis of lupus nephritis and holds promise as a therapeutic target (46). Literature reports on SPOCK2 have mostly focused on its role in tumorigenesis and progression. It has been reported that the has-miR-363-3p-SPOCK2 axis is involved in regulating the cytoskeleton of actin cells and plays a regulatory role in the staging and progression of ovarian cancer (47). SPOCK2 downregulation significantly inhibits proliferation and invasion of OC cells while promoting cell apoptosis (48). In patients with pulmonary adenocarcinoma, SPOCK2 expression is downregulated, while high expression of SPOCK2 prolongs the survival of LUAD patients. Further investigation into the molecular mechanisms of SPOCK2’s role in LUAD revealed that this could be partially due to its association with tumor-infiltrating immune cells (49). Interestingly, PHF13 (also known as SPOC1) differs from the four previously mentioned proteins in that its function primarily focuses on DNA damage repair and chromatin structure regulation (50), involving chromatin binding and histone methylation binding, and is associated with the development of ovarian cancer (51). AGRN, a proteoglycan, is one of the core components of the BM structure. It is expressed widely in any tissue and is especially important for the formation, maintenance, plasticity, and signal transmission of synapses in the central nervous system (52). Mice with mutations that result in the absence of AGRN expression exhibit nonfunctional neuromuscular junctions and suffocate to death *in utero* or shortly after birth (53). Exploring the underlying molecular mechanisms reveals that AGRN is essential for the formation, maintenance, and regeneration of neuromuscular junctions through the LRP4/MuSK pathway (54, 55). In adult hippocampus, genetic deficiency of AGRN reduces the proliferation of neural stem/progenitor cells (NSPCs) and increases depression-like behavior (56). In clinical practice, about 40-90% of SLE patients present with neuro-psychiatric manifestations such as depression, cognitive impairment, and psychosis, known as neuropsychiatric systemic lupus erythematosus (NPSLE) (57). The AGRN expression in the brain tissue of SLE and its impact on the progression of NPSLE requires further investigation. In addition, AGRN is a crucial regulatory factor in the epithelial-mesenchymal transition in epicardium, promoting epicardial cell proliferation (58, 59), reducing myocardial ischemia-reperfusion injury and improving cardiac function (60).

We constructed a diagnostic model for SLE based on the feature genes, and calibration curve and decision curve analysis demonstrated a good fit of the model, with an AUC of 0.955 for the diagnostic model. Subsequent ROC analysis revealed that AGRN exhibited the highest value. This highlights the potential diagnostic value of AGRN in SLE. According to GSEA analysis, the AGRN high-expression subgroup was enriched for the signaling pathways for the B cell receptor and chemokines. By immune infiltration analysis, the SLE group had higher levels of aDCs and Treg infiltration than healthy controls, and immune functions such as APC co-inhibition, inflammation promotion, MHC-I, parainflammation, and type I IFN response were stronger in the SLE group than in the healthy controls. AGRN was significantly correlated with the above immune functions. This is consistent with existing research findings indicating that AGRN has a significant impact on the immune system. The expression of AGRN in T cells has long been confirmed. Khan reported the expression of AGRN in mouse thymocytes and splenocytes (61). AGRN participates in the activation of T lymphocytes by binding to α-DG protein to promote the formation of immunological synapses between T cells and target cells (62, 63), while AGRN is post-translationally modified after T cell activation. The addition of purified AGRN from activated T cells to the medium of resting T cells induces the aggregation of lipid rafts and TCRs (61). Another study has shown that AGRN is mainly expressed in monocytes, while its expression in lymphocytes and granulocytes is significantly lower. The cell-autonomous signal transmitted by AGRN is perceived by macrophages via α-DG receptor, which facilitates cytoskeletal rearrangement during synapse formation and phosphorylation of Erk1/2 (64). SLE patients produce type I interferon, which can shift initial CD4 T cells from Th1 subtype to the predominant Tfh cell phenotype, promoting B cell differentiation, immunoglobulin class switching, and ultimately leading to secretion of anti-nuclear antibodies (ANA) (65, 66). Based on bioinformatics analysis, we have demonstrated a significant correlation between AGRN and IFN-I in SLE patients, suggesting AGRN may have potential synergistic effects on IFN-I-mediated T lymphocyte differentiation and activation. The basement membrane, as the main component of the extracellular matrix, guides cell polarity, differentiation, and migration, and plays an important role in tumor progression. Therefore, we selected the basement membrane related gene AGRN for pan-cancer analysis.

It has been reported that malignant tumors are a potential complication in SLE patients (67). The population with SLE has unique cancer risk characteristics. Compared with the general population, SLE patients had a higher chance of developing 24 site-specific tumors, particularly non-Hodgkin lymphoma (68, 69). The risk of melanoma, prostate cancer, and breast cancer is reduced, while there is no difference in the risk of 11 other malignancies (9, 70). However, the mechanisms underlying the association between SLE and cancer remain elusive. Excessive stimulation of B cells and defects in immune system surveillance systems during SLE are considered one of the reasons for increased cancer risk (68). In addition, the interaction between medication exposure and virus exposure in SLE patients, although currently unconfirmed, is also considered another risk factor for increased cancer incidence (71, 72). Recent research has shown that AGRN is upregulated in various tumor tissues (73–76), and plays an important role in regulating malignancy development and immune microenvironment (73, 77, 78). This may be one of the reasons why SLE patients have a higher risk of certain tumors compared to healthy individuals (79, 80). Our research indicates that AGRN expression is significantly higher in the majority of tumor tissues. Furthermore, AGRN expression is associated with the pathological staging of COAD, HNSC, KIRC, LIHC, PAAD, and TGCT. Overall, this suggests that AGRN holds promising prospects for cancer diagnosis.

AGRN expression levels affect the prognosis of tumor patients. High AGRN expression was a high-risk factor for LIHC, PAAD, SARC, and PRAD, while high AGRN expression indicated a significantly better prognosis for BRCA. Analysis of tumor immune cell infiltration revealed that AGRN is closely associated with memory CD4+T cells activated, monocytes, plasma cells, Tfh cells, NK cells activated, mast cells resting, and DCs resting. It is noteworthy that AGRN expression is positively correlated with multiple subtypes of infiltrating macrophages (M0, M1, and M2). Overall, our findings suggest that AGRN may play a part in promoting tumorigenesis and act as a potential cancer prognosis biomarker.

Although our research has shown that the BM-related genes AGRN plays a big part in the progression of SLE and multiple malignancies, the upstream and downstream molecular regulatory mechanisms of AGRN in SLE have not been demonstrated *in vivo*. In addition, we conducted a diagnostic efficiency analysis of AGRN, and found a positive correlation between AGRN and disease activity, but without statistical significance. This is related to a small sample size and inconsistent activity scores and sampling time for some patients. Therefore, it is necessary to further evaluate the correlation between AGRN and disease activity, explore the specificity and common molecular mechanisms of AGRN in the progression of SLE and tumors, particularly hepatocellular carcinoma, should be conducted, which may provide new insights for personalized medicine.

## Conclusion

In our research, we have examined the level of BM-related genes in SLE. AGRN was identified as a key molecular biomarker in the etiopathogenesis of SLE, which may offer more latent therapeutic targets for clinical treatment. However, it is necessary to validate and develop this discovery through further research. In pan-cancer analysis, the expression levels of AGRN vary in different kinds of tumors and may become an independent prognostic factor for certain tumors. In summary, AGRN is considered the most promising target in the development of SLE and multiple tumors, which may bring hope for the treatment of human immune-related diseases.

## Data availability statement

Publicly available datasets were analyzed in this study. This data can be found here: https://www.ncbi.nlm.nih.gov/geo/query/acc.cgi?acc=GSE110169, https://www.ncbi.nlm.nih.gov/geo/query/acc.cgi?acc=GSE185047.

## Ethics statement

The studies involving humans were approved by the Medical Ethics Expert Committee of Zibo Central Hospital. The studies were conducted in accordance with the local legislation and institutional requirements. The participants provided their written informed consent to participate in this study.

## Author contributions

RL and XZ researched the article and wrote the manuscript. JG and LD processed the images. CF and JH assisted in the processing of the datasets. LD and JS reviewed and edited the manuscript before submission. All authors contributed to the article and approved the submitted version.
