# Supplementary material for: Bioinformatics-based analysis of the roles of basement membrane-related gene AGRN in systemic lupus erythematosus and pan-cancer development
Source: Front Immunol. 2023 Sep 29;14:1231611. doi: 10.3389/fimmu.2023.1231611 (PMC10570813; doi:10.3389/fimmu.2023.1231611)
Supplement: Supplementary file 1 [file DataSheet_1.docx]

Supplementary Table 1. Primer sequences

| Gene | Sequences |
| --- | --- |
| *Agrn* | Forward: 5'-GGTGGGTCTCATCTTTCCCA-3' |
|  | Reverse: 5'-ACGGGACGTTGCAGTTTAAG-3' |
| *Gapdh* | Forward: 5'- CCGCATCTTCTTTTGCGTCG-3' |
|  | Reverse: 5'- ATCCGTTGACTCCGACCTTC-3' |

Supplementary Figure 1


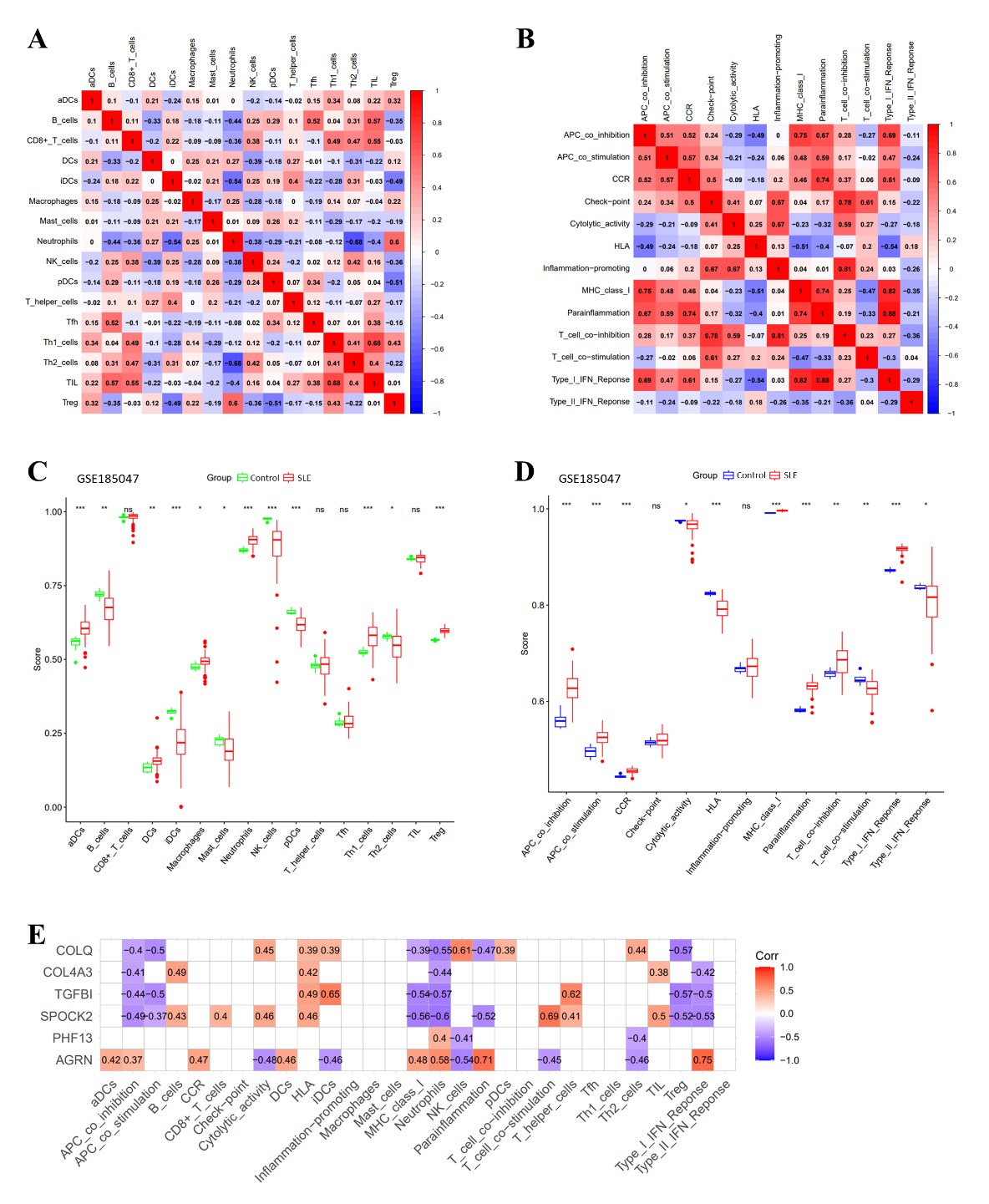


**Supplementary Figure 1.** Analysis of ssGSEA immune infiltration (*validation queue GSE185047 dataset*). **(A)** Correlation analysis between immune cells; **(B)** Correlation analysis between immune functions; **(C)** Boxplot of differences in immune cell infiltration between SLE and healthy controls; **(D)** Boxplot of immune function differences between SLE and healthy controls; **(E)** Correlation analysis of feature genes with immune function. (ns: no statistical difference, **p*<0.05, ***p*<0.01, ****p*<0.001).

Supplementary Figure 2


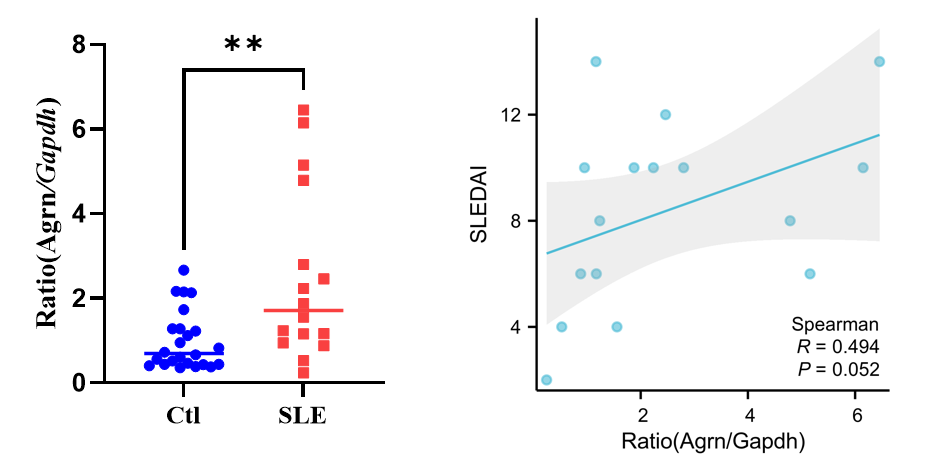


**Supplementary Figure 2.** Experimental verification of AGRN expression in whole blood of SLE patients. **(A)** Determination of AGRN mRNA content in peripheral blood; **(B)** Correlation analysis of AGRN and SLEDAI scores. (***p*<0.01).

Supplementary Figure 3


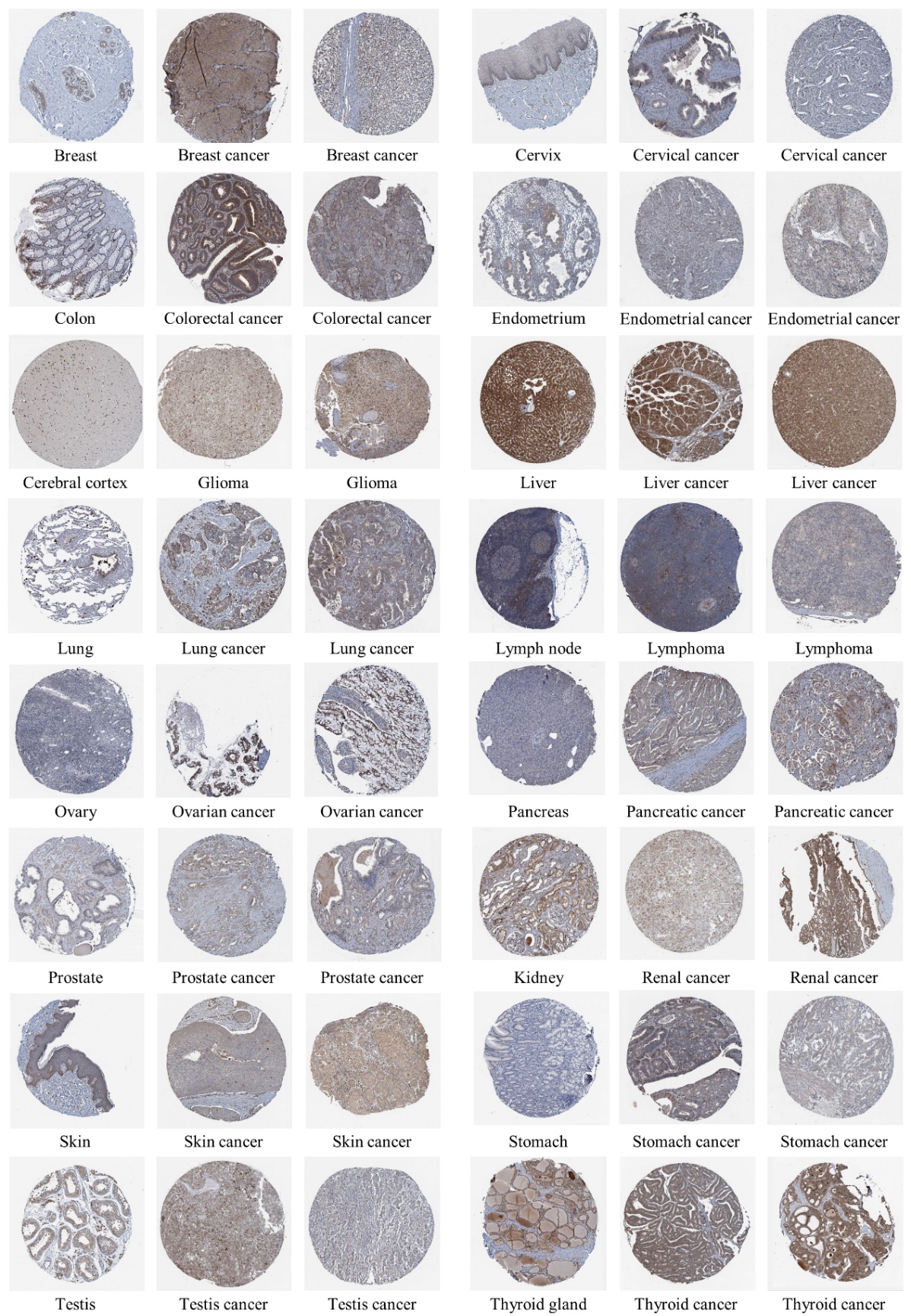


**Supplementary Figure 3.** The protein expression of AGRN in immunohistochemical images of normal (left) and tumor (right) groups
